# Ultra-Small Superparamagnetic Iron-Oxide Nanoparticles Exert Different Effects on Erythrocytes in Normotensive and Hypertensive Rats

**DOI:** 10.3390/biomedicines9040377

**Published:** 2021-04-02

**Authors:** Jana Radosinska, Tomas Jasenovec, Dominika Radosinska, Peter Balis, Angelika Puzserova, Martin Skratek, Jan Manka, Iveta Bernatova

**Affiliations:** 1 Institute of Physiology, Faculty of Medicine, Comenius University in Bratislava, 813 72 Bratislava, Slovakia; tomas.jasenovec@fmed.uniba.sk; 2 Centre of Experimental Medicine, Institute for Heart Research, Slovak Academy of Sciences, 841 04 Bratislava, Slovakia; 3 Department of Molecular Biology, Faculty of Natural Sciences, Comenius University in Bratislava, 842 15 Bratislava, Slovakia; dominikaradosinska@gmail.com; 4 Centre of Experimental Medicine, Institute of Normal and Pathological Physiology, Slovak Academy of Sciences, 813 71 Bratislava, Slovakia; peter.balis@savba.sk (P.B.); angelika.puzserova@savba.sk (A.P.); iveta.bernatova@savba.sk (I.B.); 5 Institute of Measurement Science, Slovak Academy of Sciences, 841 04 Bratislava, Slovakia; martin.skratek@savba.sk (M.S.); jan.manka@savba.sk (J.M.)

**Keywords:** iron-oxide nanoparticles, SPIONs, erythrocytes, plasma, deformability, nitric oxide, osmotic resistance, hypertension, biomagnetometry

## Abstract

We determined erythrocyte physiological and biochemical properties after the single and repeated administration of ultra-small superparamagnetic iron-oxide nanoparticles (USPIONs) in normotensive Wistar–Kyoto (WKY) and spontaneously hypertensive (SHR) rats. Polyethylene glycol-coated USPIONs (transmission electron microscope detected a mean size of ~30 nm and hydrodynamic size ~51 nm) were intravenously administered to rats either in one infusion at nominal dose 1 mg Fe/kg or in two infusions (administered with a difference of 24 h) at nominal dose 2 mg Fe/kg. Results showed that USPIONs did not deteriorate erythrocyte deformability, nitric oxide production, and osmotic resistance in both experimental settings. Both the single and repeated USPION administration elevated erythrocyte deformability in WKY. However, this effect was not present in SHR; deformability in USPION-treated SHR was significantly lower than in USPION-treated WKY. Nitric oxide production by erythrocytes was increased after a single USPION treatment in WKY, so it can be associated with improvement in erythrocyte deformability. Using biomagnetometry, we revealed significantly lower amounts of USPION-originated iron in erythrocytes in SHR compared with WKY. We found a much faster elimination of USPIONs from erythrocytes in hypertensive rats compared with the normotensive ones, which might be relevant for clinical practice in hypertensive patients undergoing clinical examination with the use of iron-oxide nanoparticles.

## 1. Introduction

Red blood cells (RBCs) are the most abundant blood elements. Their main task is to secure the transport of respiratory gases from lungs to tissues and vice versa. In addition to this well-known function, RBCs also participate in the regulation of vascular tone, redox balance [1], hemostasis, and thrombosis [2]. The property allowing RBCs to continuously circulate in different segments of blood circulation is called deformability. More precisely, RBC deformability represents their ability to change the shape depending on blood flow without sustaining damage. This property is crucial when RBCs are passing through capillaries with a smaller diameter than the diameter of RBC itself and could also be the major determinant of impaired perfusion and occlusion in microvessels [3,4]. Additionally, RBC deformability is responsible for non-Newtonian behavior of blood, thus lowering blood viscosity in large vessels [5]. RBC deformability is determined by many factors, such as maintaining the ionic homeostasis or the production of nitric oxide (NO) by RBCs themselves [3,6,7,8]. It is possible as RBCs possess functional endothelial NO synthase. Consequently, RBCs do not only serve as scavengers of endothelium-produced NO, but significantly contribute to the total pool of NO in the bloodstream and participate in the regulation of blood flow in the tissues [9].

Wistar–Kyoto (WKY) rats and spontaneously hypertensive rats (SHR) are two of the most commonly used experimental rat strains. SHRs are used as a model for human essential hypertension, and they are often compared with WKY rats that represent healthy controls in the majority of studies. Mechanisms involved in hypertension development and hypertension per se affect various tissues as well as RBCs. Male SHR rats have a higher RBC count as well as elevated hematocrit than WKY rats [10]. Another study showed increased RBC aggregability and plasma hyperviscosity in SHR even during the early stage of hypertension development [11]. It is documented that SHRs have impaired RBC deformability in comparison with WKY rats [12]. Increased membrane tubulin content seems to play an important role in the modulation of RBC deformability in SHR [13]. RBCs from SHR tend to have altered antioxidant enzymes and increased susceptibility to in vitro lipid oxidation [14]. In addition, the RBC membrane of SHR is also more permeable to Ca^2+^, Na^+^, and K^+^ in comparison with WKY rats. [15,16]. Considering these differences between normotensive and hypertensive subjects, SHRs were used in this study to investigate the effects of superparamagnetic iron oxide nanoparticles (SPIONs) on the fundamental properties of RBCs in SHR. SPIONs are generally considered as biocompatible, biodegradable, and non-toxic nanoparticles that are used in a wide range of applications in medicine, including magnetic resonance imaging, magnetic hyperthermia, cell labeling, photothermal therapy, and drug delivery [17,18,19]. The toxicity of iron oxide nanoparticles depends on their dose and type and can be greatly lowered by using biocompatible nanoparticle coating [17,19]. Most SPIONs are therefore coated with silicon, dextran, citrate, or polyethylene glycol (PEG). PEG is a stable hydrophilic polymer considered biocompatible, which prevents rapid elimination of SPIONs from circulation [20]. Nevertheless, it was suggested that nanoparticle administration might lead to cell damage; however, while using PEG-coated nanoparticles, no signs of hemolysis, genotoxic effects, or modulation of erythropoiesis were detected in female Wistar rats. PEG-coated nanoparticles do not have an impact on leukocyte count; thus are preferred [21]. However, PEG was shown to have an immunogenic effect, which may lead to faster clearance and lower target tissue accumulation [22]. Magnetite nanoparticles coated with PEG did not show cytotoxic effect in a culture of mesenchymal stem cells at concentrations from 10 to 1000 µg/mL or a hemolytic effect in human RBCs at concentrations of 100 µg/mL ex vivo [23]. In biomedical applications, intravenous (i.v.) administration of SPIONs is the common route for their applications. In humans, iron oxide nanoparticles, when administered as contrast agents to improve magnetic resonance imaging, induced vasodilatation and hypotension [24]. In our previous study, PEGylated SPIONs did not affect blood pressure in rats; however, they increased the NO-dependent component of acetylcholine-induced relaxation in the femoral arteries and had tissue-dependent effects on NO synthase activity [25]. After intravenous administration, nanoparticles are transported via the bloodstream; thus, the interaction of nanoparticles with RBCs can alter RBC function during in vivo conditions. The extent of these interactions may depend on the dose and physico-chemical properties of nanoparticles as well as on the duration of nanoparticle presence in the circulation. We found SPION-originated iron in the tissues of the left heart ventricle, aorta, liver, and kidneys when administered intravenously; however, the highest levels of nanoparticle-originated iron were found in blood [26]. For this reason, we further focused on RBC properties.

With the aim to reveal the influence of SPIONs on RBCs in conditions of high blood pressure as well as to understand better the effect of acute and repeated i.v. SPION administration, we have determined RBC properties such as deformability, NO production, and osmotic resistance after the administration of PEGylated ultra-small SPIONs (USPIONs) in two experimental designs: (1) we studied the short-term effects of acute USPION administration on RBC properties as well as (2) the effect of repeated administration of USPIONs 24 h post-treatment. In addition, we focused on the potentially different influence of USPIONs on RBCs in SHR compared with normotensive WKY rats due to the above-mentioned differences in RBC properties in hypertensive subjects. RBC properties were determined together with the investigation of the multiple biochemical parameters in blood plasma.

## 2. Materials and Methods

### 2.1. Experimental Design

Normotensive WKY and spontaneously hypertensive male rats 12–16 weeks old were used in this study. All rats were housed in the certified animal facility of the Institute of Normal and Pathological Physiology, Centre of Experimental Medicine, Slovak Academy of Sciences, in order to keep the standardized environmental background for all animals. They were fed with pelleted chow for young rats Altromin 1314 (Altromin Spezialfutter, Lage, Germany) until the ninth week of age. Afterward, they were fed with Altromin 1324 chow (for adult rats). Both chows were deficient in phytoestrogens (variant P), with an iron content of 192.51 mg/kg. The food and water were available ad libitum. All rats were housed under standard conditions at 22–24 °C in a 12 h light/dark cycle.

Commercially available PEG-coated USPIONs were used in this study. USPIONs were purchased from Sigma-Aldrich (Bratislava, Slovakia, cat. No. 747408, PubChem SID 329765832, accessed on 18 March 2021). Iron content was 1 mg Fe/mL, nanoparticle concentration dispersed in water was 0.034 nmol/mL. The size of USPIONs confirmed by the transmission electron microscope was 28–32 nm, the zeta potential was −12 mV, the polydispersity index was 0.1, and the hydrodynamic size was about 45 nm (all parameters declared by the manufacturer). USPIONs were autoclaved at 121 °C for 30 min, and their exact properties were determined and published in detail in an earlier publication [26].

Two sets of experiments were performed—Experiment 1: single i.v. administration of USPIONs at the dose of 1 mg Fe/kg, and Experiment 2: repeated i.v. administration of USPIONs at the dose of 2 mg Fe/kg/day for two consecutive days.

### 2.2. Experiment 1 (Single Administration)

Rats had implanted two catheters, one in the carotid artery (for blood collection) and in the jugular vein for USPION (or saline in the controls) infusion. The catheters were implanted under anesthesia (2.5–3.5% isoflurane) using the procedure described by Liskova et al. [25]. The rats were divided into control WKY rats, USPION-treated WKY rats, control SHR rats, and USPION-treated SHR rats (*n* = 6–7 per group). USPIONs were dispersed with sterile saline to reach a final dose of 1 mg of Fe/kg of body weight in a final volume of 1 mL and infused during 10 min infusion using an infusion pump. Sterile saline (1 mL) was infused to control rats. To analyze RBC deformability and NO production by RBCs, blood samples were taken into heparin solution from the carotid artery before the beginning of the experiment and approximately 100–102 min after the end of USPION or saline infusion. Rats were subsequently sacrificed by decapitation after the exposure CO_2_ (until the loss of consciousness), and trunk blood was collected into heparin tubes for biochemical analyses and determination of USPION content in RBCs.

### 2.3. Experiment 2 (Repeated Administration)

In the second experiment, rats had implanted the catheter into the jugular vein for the i.v. infusion of USPIONs or saline in the controls. The catheter was implanted one day before the experiment in the same way as in Experiment 1.

The rats were divided into control WKY, USPION-treated WKY, control SHR, and USPION-treated SHR (*n* = 6–8 per group). USPIONs were administered at a dose of 2 mg Fe per kg of body weight per day over two consecutive days. USPIONs were dispersed in saline to a final volume of 1 mL and infused during 10 min infusion using an infusion pump. Sterile saline (1 mL) was infused to control rats. Rats were killed 24 h after the second infusion by decapitation after the exposure CO_2_ (until the loss of consciousness), and trunk blood was collected for all further analyses.

All the procedures used in this study were approved by the State Veterinary and Food Administration of the Slovak Republic (protocol code: 3619/16-221 and date of approval: 28 October 2016) in accordance with the European Union Directive 2010/63/EU.

### 2.4. RBC Deformability

In both Experiment 1 and 2, whole blood was centrifuged for 10 min (850× *g*, 4 °C). The pellet containing RBCs was washed three times using saline, while the supernatant was discarded. Resultant washed erythrocyte mass was diluted in Cellpack solution (diluent for Sysmex blood analyzer, 1:1000, *v*:*v*) and centrifuged at 175 g through membrane filters with 5 μm pores in diameter (Ultrafree-MC SV Centrifugal Filter; Merck Millipore Ltd., Tullagreen Carrigtwohill, Ireland). RBC deformability was calculated as the percentage of RBCs that passed through a centrifugal filter as published previously [27]. In Experiment 1, we measured RBC deformability before USPION administration as well as approximately 100 min after this intervention. In order to compare the response between the control and USPION-treated groups, we calculated the difference in RBC deformability in both according to the formula:

Δ deformability = (RBC deformability after USPION administration)–(RBC deformability before this intervention).

In Experiment 2, we measured RBC deformability using trunk blood taken at the end of the experiment (i.e., 24 h after the last intervention).

### 2.5. NO Production by RBCs

4,5-diaminofluorescein diacetate (DAF-2 DA) was used as an indicator of NO presence. Measurements were executed as described previously [8]. Briefly, the whole blood was diluted 1:9 in modified saline solution (in mmol/l: NaCl 118.99, KCl 4.69, NaHCO_3_ 25, MgSO_4_.7H_2_O 1.17, KH_2_PO_4_ 1.18, CaCl_2_.2H_2_O 2.5, Na_2_EDTA 0.03, glucose 5.5, and pH 7.4), treated with DAF-2 DA (25 μmoL/L, Abcam, Cambridge, UK) and incubated at room temperature in the dark for 10 min. NO related fluorescence was detected by fluorescence microscope (Nikon Eclipse Ti, Tokyo, Japan) using filters for fluorescein isothiocyanate (λex. 465–495 nm and λem. 515–555 nm). For quantification and evaluation of fluorescence signal, ImageJ software was used. RBCs passing through image edges, overlapping RBCs, and non-RBC particles were discarded. Presented values represent an average fluorescence of a single RBC in integrated density units (the sum of the pixels’ luminous intensities). In Experiment 1, we measured NO production by RBCs before USPIONs administration as well as approximately 100 min after this intervention. In order to compare the response between the control and USPIONs groups, we calculated the difference in NO production by RBCs in both according to the formula:

Δ NO production = (NO production by RBCs after USPION administration)–(NO production by RBCs before this intervention).

In Experiment 2, we measured NO production by RBCs using trunk blood taken at the end of the experiment.

### 2.6. Osmotic Resistance of RBC

Osmotic resistance of RBCs was determined only in Experiment 2 (repeated USPIONs administration). RBC mass was diluted in solutions with various concentrations of NaCl (0.1%–0.9%). Samples were mixed, then incubated for 30 min at room temperature, and centrifuged at 800× *g* for 5 min. Hemolysis intensity in supernatants was determined spectrophotometrically at 540 nm. Supernatant corresponding to 0.9% NaCl solution was adjusted to 0% hemolysis, and supernatant corresponding to 0.1% NaCl was adjusted to 100% hemolysis. Obtained data were interpolated to a sigmoidal curve and used to calculate the IC_50_, i.e., the NaCl concentration at which 50% hemolysis occurred.

All chemicals used in this study were purchased from Sigma-Aldrich, Bratislava, Slovakia, unless otherwise stated.

### 2.7. Determination of the USPION-Originated Iron Content in RBCs

Isolated RBCs were placed in the Eppendorf test tubes and stored at −80 °C. Subsequently, the RBCs were defrosted and homogenized using an ultrasonic bath for 60 s (50 kHz and 30 W) [28]. After homogenization, 20 μL of RBCs were vacuum dried for 1 h, placed into the plastic straw, and their hysteresis curves (magnetization *M* vs. applied field *H* dependence) were measured at the temperature of 300 K and up to 1 T magnetic field as described previously [26]. The USPION-originating iron content in the RBCs was determined using Equation (1)

(1) $$c\left[\frac{\mu g_{Fe}}{g}\right]=\frac{M_{s}^{\prime }\;*\;m_{Fe}}{M{\;}_{USPIONs}\;*\;m_{s}}*10^{6}$$

where *c* is the USPION-originated Fe content in the sample, *M’_S_* is sample magnetization (at 1 T) after subtraction of magnetization of the control tissue, *m_s_* is the mass of the sample, *m_Fe_* is the mass of iron in 10 µL of USPIONs, and *M_USPIONs_* is the magnetization of USPION dispersion measured at temperature 300 K and magnetic field 1 T. A Quantum Design MPMS-XL 7AC (San Diego, CA, USA) SQUID (superconducting quantum interference device) magnetometer with a reciprocating sample operating option was used for measurements.

### 2.8. Biochemical Parameters

Biochemical parameters (concentrations of iron, calcium, potassium, magnesium, total cholesterol, triglycerides, uric acid, creatinine, inorganic phosphate, total proteins, albumins, and urea; activities of alkaline phosphatase, alanine transaminase, aspartate transaminase, and lactate dehydrogenase) were determined in blood plasma from heparinized trunk blood (centrifugation at 4 °C, 850 *g*, 10 min) in the accredited laboratory of Laboklin s.r.o. (Bratislava, Slovakia) using the standard laboratory methods.

### 2.9. Statistical Analyses

Statistical analysis was performed by two-way ANOVA for repeated measures (time of measurement, i.e., before vs. after USPION administration in Experiment 1 or two-way ANOVA (with intervention and rat strain as the independent factors) where appropriate. ANOVA analyses were followed with Bonferroni’s post-hoc test or Tukey’s multiple comparison test. Outliers were detected using the Grubbs’ test and removed from further analyses. The values were found to significantly different when *p* < 0.05. The data are presented as mean ± standard deviation (SD). GraphPad Prism v7.02 (GraphPad Software, Inc., San Diego, CA, USA) and Statistica v13.5 (StatSoft Europe, Hamburg, Germany) were used for the statistical analyses.

## 3. Results

### 3.1. Experiment 1

#### 3.1.1. RBC Deformability

**Figure 1:**
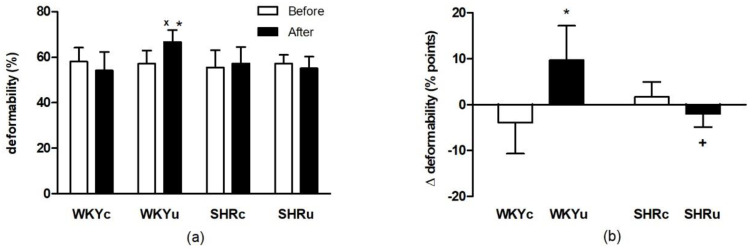
Red blood cell deformability determined before and after the single USPION administration at a dose of 1 mg Fe/kg body weight (**a**) and the difference in red blood cell deformability measured after the USPIONs administration and before it (**b**). Rats were killed approximately 100 min after the USPIONs administration. Abbreviations: USPIONs, ultra-small superparamagnetic iron-oxide nanoparticles; WKY, Wistar Kyoto rats; SHR, spontaneously hypertensive rats; WKYc, control WKY; WKYu, USPION-treated WKY; SHRc, control SHR; SHRu, USPION-treated SHR. The data are presented as means ± SD, *n* = 6–7 per group. * *p* < 0.05 vs. WKYc (in corresponding time point); ^x^ *p* < 0.05 vs. WKYu before USPION administration; ^+^ *p* < 0.05 vs. WKYu.

Evaluation of RBC deformability by two-way ANOVA for repeated measures revealed a significant strain × intervention × time interaction (F_(1,20)_ = 12.56; *p* < 0.005; *n* = 24). There was a significant elevation of RBC deformability in USPION-treated WKY rats vs. the levels determined before the intervention in this group (*p* < 0.01) as well as compared with the control WKY rats after saline infusion (*p* < 0.05). Such changes were not found in SHR rats (Figure 1a).

Two-way ANOVA also revealed significant effect of strain and intervention interaction (F_(1,20)_ = 12.56; *p* < 0.005, *n* = 24) in the differences in RBC deformability values measured after USPION administration and before it (Figure 1b).

#### 3.1.2. NO Production in Red Blood Cells

**Figure 2:**
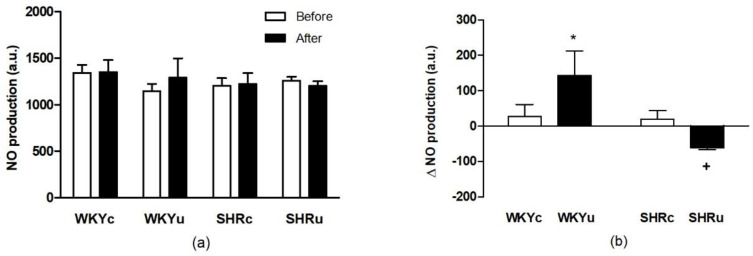
Nitric oxide production by red blood cells determined before and after the single USPIONs administration at tahe dose of 1 mg Fe/kg body weight (**a**) and the difference in nitric oxide production by red blood cells measured after the USPIONs administration and before it (**b**). Rats were killed approximately 100 min after the USPION administration. Abbreviations: NO, nitric oxide; USPIONs, ultra-small superparamagnetic iron-oxide nanoparticles; WKY, Wistar Kyoto rats; SHR, spontaneously hypertensive rats; WKYc, control WKY; WKYu, USPION-treated WKY; SHRc, control SHR; SHRu, USPION-treated SHR. The data are presented as means ± SD, *n* = 6–7 per group; * *p* < 0.05 vs. WKYc; ^+^ *p* < 0.05 vs. WKYu.

The evaluation of NO production by RBCs by two-way ANOVA for repeated measures did not reveal a significant difference among the groups before and after treatment (Figure 2a). However, the calculation of the difference in NO production before and after USPION administration revealed significantly higher NO production in USPION-treated WKY rats compared with the USPION-treated SHR rats (Figure 2b, *p* < 0.05).

#### 3.1.3. Total Iron Content in Plasma and USPION-Originated Iron Content in RBCs

**Figure 3:**
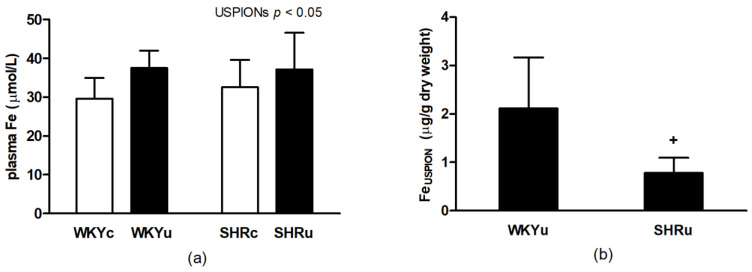
Total iron content in plasma (**a**) and USPION-originated iron content in RBCs (**b**) after the single USPION administration at a dose of 1 mg Fe/kg body weight. Rats were sacrificed approximately 100 min after the USPION administration. Abbreviations: RBCs, red blood cells; USPIONs, ultra-small superparamagnetic iron-oxide nanoparticles; WKY, Wistar Kyoto rats; SHR, spontaneously hypertensive rats; WKYc, control WKY; WKYu, USPION-treated WKY; SHRc, control SHR; SHRu, USPION-treated SHR. The data are presented as means ± SD, *n* = 6–7 per group (**a**) and *n* = 5 per group; (**b**) ^+^ *p* < 0.05 vs. WKYu.

Two-way ANOVA revealed significant main effect of intervention on total iron content in plasma (F_(1,21)_ = 5.23; *p* < 0.05; *n* = 25), with higher values in USPION-treated rats (Figure 3a). USPION-originated iron content in fraction of RBCs was significantly lower in SHR compared with WKY rats (*p* < 0.05) (Figure 3b).

#### 3.1.4. Biochemical Analyses

Biochemical analysis of plasma (Table 1) showed that there were no differences in the calcium, potassium, and magnesium ion concentration among the experimental groups. There were strain-dependent differences only in sodium and total cholesterol concentrations, which both were decreased in SHR compared with WKY rats (Table 1).

The significant main effect of the intervention was found in triglyceride concentration, with higher levels found in USPION-treated animals. The significant interaction of strain and intervention was revealed only in alkaline phosphatase activity, with higher levels in USPION-treated SHR rats compared with control SHR rats and lower levels in USPION-treated WKY rats when compared with control WKY rats. All biochemical parameters measured in plasma of rats, including the two-way ANOVA results, are summarized in Table 1.

### 3.2. Experiment 2

#### 3.2.1. RBC Deformability

**Figure 4:**
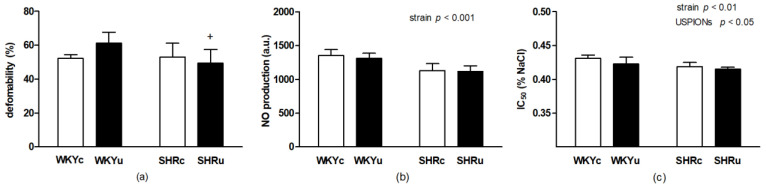
The effect of repeated USPION administration at the dose of 2 mg Fe/kg of body weight per day on RBC properties—deformability (**a**), nitric oxide production (**b**), and osmotic resistance (**c**). Rats were killed approximately 24 h after the second USPION administration. Abbreviations: USPIONs, ultra-small superparamagnetic iron-oxide nanoparticles; WKY, Wistar Kyoto rats; SHR, spontaneously hypertensive rats; WKYc, control WKY; WKYu, USPION-treated WKY; SHRc, control SHR; SHRu, USPION-treated SHR; IC_50_, NaCl concentration at which 50% hemolysis occurred. Results are presented as mean ± SD, *n* = 6–7 per group. ^+^ *p* < 0.05 vs. WKYu.

In the second experiment–in repeated USPION administration, two-way ANOVA revealed a significant main effect of strain (F_(1,22)_ = 4.34; *p* < 0.05; *n* = 26) with higher levels of RBC deformability found in WKY rats vs. SHR rats (Figure 4a). In addition, RBC deformability was statistically different among the groups as the interaction of strain with the intervention was significant (F_(1,22)_ = 5.58; *p* < 0.05; *n* = 26) after the repeated USPION administration. There was a significantly higher RBC deformability in USPION-treated WKY rats vs. USPION-treated SHR rats (*p* < 0.05, Figure 4a).

#### 3.2.2. RBC NO Production

Regarding NO production after repeated USPION treatment, ANOVA revealed a significant main effect of strain (F_(1,22)_ = 35.58; *p* < 0.0001; *n* = 26) with significantly higher levels of NO found in WKY rats vs. SHR rats (*p* < 0.0001, Figure 4b). No effect of USPIONs or interaction were found.

#### 3.2.3. RBC Osmotic Resistance

Regarding the RBC osmotic resistance, the main effect of strain (F_(1,23)_ = 14.60; *p* < 0.001; *n* = 27) was significant and a higher osmotic resistance was found in SHR rats vs. WKY rats (p < 0.005). There was also significant main effect of intervention (F_(1,23)_ = 5.51; *p* < 0.05; *n* = 26), USPION administration led to significantly increased osmotic resistance compared with control rats (*p* < 0.05, Figure 4c).

#### 3.2.4. Total Iron Content in Plasma and USPION-Originated Iron Content in RBCs

**Figure 5:**
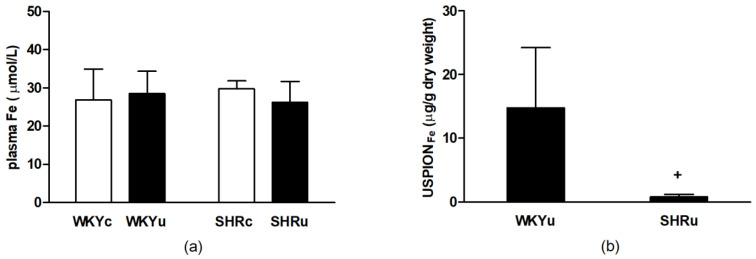
The effect of repeated USPIONs administration at the dose of 2 mg Fe/kg of body weight per day on total iron concentration in blood plasma (**a**) and USPION-originated iron content in RBCs (**b**). Rats were killed approximately 24 h after the second USPIONs administration. Abbreviations: USPIONs, ultra-small superparamagnetic iron-oxide nanoparticles; WKY, Wistar Kyoto rats; SHR, spontaneously hypertensive rats; WKYc, control WKY; WKYu, USPION-treated WKY; SHRc, control SHR; SHRu, USPION-treated SHR. Results are presented as mean ± SD, *n* = 6–7 per group (**a**), and *n* = 5 per group (**b**). ^+^ *p* < 0.05 vs. WKYu.

There were no differences in total iron content in plasma among the groups (Figure 5a). USPION-originated iron content in a fraction of RBCs of rats exposed to USPIONs repeatedly was significantly lower in SHR rats compared with WKY rats (*p* < 0.01) (Figure 5b).

#### 3.2.5. Biochemical Analyses

Biochemical analysis of plasma (Table 2) showed that there were no differences in the concentration of calcium, potassium, magnesium, or sodium ions among the experimental groups. There were strain-dependent differences in total cholesterol, triglyceride, and inorganic phosphate concentrations, which were decreased in SHR rats compared with WKY rats (Table 2). In addition, strain-dependent differences in alkaline phosphatase, uric acid, and albumins were observed, which were elevated in SHR rats vs. WKY rats. Regarding the intervention dependent differences, USPION administration led to a decrease in uric acid, creatinine, and albumin concentration in the blood plasma of experimental animals. A significant interaction of both factors was found only for uric acid concentration. All biochemical parameters measured in plasma of rats, including the two-way ANOVA results, are summarized in Table 2.

## 4. Discussion

The investigation of RBC-related effects of nanoparticles is actually and continuously relevant as RBCs are studied as potential carriers of USPIONs in the blood to prevent their elimination from the organism and to keep them in a concentration sufficient for the long-term magnetic resonance imaging [29,30]. RBCs are also used to evaluate the efficacy and toxicity of various nanoparticles, mostly under in vitro and ex vivo conditions using hemolytic assay or RBC aggregation [31,32]. Regarding the effect of USPIONs on RBCs, we have determined more complex parameters of RBCs—their deformability, in two experimental designs—in single and repeated nanoparticle administration. In addition, we used two rat strains or genotypes—normotensive rats and rats representing the animal model of human essential hypertension—SHR. Our results showed that PEGylated Fe_3_O_4_ nanoparticles did not deteriorate RBC deformability either after single or repeated USPION treatment in both genotypes. Moreover, a single infusion of USPIONs elevated RBC deformability in WKY rats. However, this effect was not present in the SHR strain. RBC deformability in USPION-treated SHR rats in both experiments was significantly lower than that of USPION-treated WKY rats. To the best of our knowledge, the different effect of USPION administration on RBCs in normotensive and hypertensive animals has not yet been observed. We hypothesize that increase in RBC deformability in normotensive rats after USPION treatment is transient and reactive, as according to some findings, USPIONs could be potentially toxic [19]. In the case of SHR rats, such response is not observable due to the potential depletion of RBC reserves. As together with improved RBC deformability, NO production was increased in RBCs of WKY rats acutely after the single USPIONs administration; we assume that changes in RBC deformability may be at least partially attributed to elevated NO production. However, NO production was unchanged in RBCs after repeated USPION administration, which could be a result of the fact that the amount of USPION was considerably reduced in blood 24 h after administration. This is supported by our findings that total iron content in plasma was elevated only after the acute USPION administration (i.e., approximately 100 min after infusion, when determined as the main effect of the intervention), while no increase was found in rats with repeatedly administered USPIONs, killed 24 h after USPION infusion, suggesting USPION elimination from blood plasma within this time interval.

Interesting results were found using SQUID biomagnetometry to determine the USPION-originating iron in RBC fraction and showed significantly lower amounts of USPION-originating iron in SHR rats in both experimental approaches. These findings point to higher elimination of nanoparticles from RBCs in SHR rats, the lower influx of USPIONs into RBCs of SHR rats and/or to reduced interactions of PEGylated USPIONs with RBC membranes in SHR rats. To the best of our knowledge, there is no other study that investigated the effect of chronically elevated blood pressure on USPION elimination, RBC deformability, and NO production. Regarding the influence of high blood pressure, we have recently found that acute stress, associated with sudden elevation of blood pressure, also reduced the amount of USPIONs in the whole blood of WKY rats [25]. Elevated blood pressure, associated with elevated blood flow, might accelerate USPION decomposition. Despite the organism lacks the regulated mechanism of iron excretion, USPIONs decomposed to nanoparticles bigger than 6 nm can be excreted via hepatobiliary clearance, while particles smaller than 6 nm can be excreted by kidneys [33]. Renal excretion seems to be plausible in conditions of acutely elevated blood pressure as our previous study showed elevated USPION-originated iron in the kidneys of acute stress-exposed WKY in line with reduced USPIONs in whole blood [25].

We also investigated changes in NO production by RBCs. NO is not only an important vasodilator and neurotransmitter, but it also plays a crucial role in the modification of RBC deformability [34]. As together with improved RBC deformability, NO production was increased in the RBCs of WKY rats after acute USPION administration; we assume that changes in RBC deformability may be at least partially attributed to elevated NO production. However, NO production was unchanged in RBCs after repeated USPION administration, which could result from the above-described changes in iron content in the blood and/or RBC fraction. In addition, the different effects of USPION administration on NO production of WKY rats and SHR rats might be caused by dysregulation of NO generation and/or elevated NO inactivation by superoxide in hypertension conditions [35]. We also observed lower RBC-derived NO production in SHR rats in comparison with WKY, which may be related to the observation of a lower influx of NO precursor–L-arginine-into RBCs in SHRs [36].

In our study, repeated USPION treatment also improved RBC osmotic resistance independently of the genotypic background of experimental animals. Interestingly, RBCs in SHR rats possessed higher resistance to hypoosmotic stress than RBCs from WKY rats. The opposite was observed in humans—RBC osmotic resistance was lower in patients with essential hypertension and normotensive subjects with a family history of hypertension, compared with normotensive controls without a family history of hypertension. However, this was not observed in patients suffering from secondary hypertension [37,38].

To determine additional effects or possible toxicity of USPIONs in normotension and hypertension that may also be related to changes in RBC properties, we determined a large variety of biochemical parameters in blood plasma. In general, the levels of biochemical parameters in rats depend on several factors such as strain, sex, age, food, and experimental protocol. No proof of acute toxicity was found in our study as the values of parameters such as LDH, AP, ALT, AST, urea, and ion concentration (Na, Ca, K, and Mg) were unaltered by USPIONs in both experimental protocols. In addition, between-strain variances were also negligible; the differences were found in the concentration of cholesterol, triglycerides, uric acid, inorganic phosphates, albumins, and AP activity. Lower plasma cholesterol and triglycerides in SHR rats, when compared with WKY rats, have already been observed [39,40]. The absence of strain-related differences in hepatic enzymes may result from the fact that young adult rats were used in our study; we may assume that differences can be found between elderly WKY rats and SHR rats, as hypertension have been linked with the development of non-alcoholic fatty liver disease [41,42,43]. After acute USPION treatment, only triglycerides were elevated (main effect of the intervention). In addition, the uric acid concentration that was higher in SHR rats in comparison with WKY rats was decreased 24 h after USPION infusion in SHR rats. It may be related to changes in oxidation status or an increase in uric acid elimination by the kidneys, which is in line with reduced creatinine and suggests increased renal excretion. Regarding the safety of USPIONs used in our experiments, we were focused mainly on RBC properties, and more studies are needed to establish their complex effect on other tissues and systems in the organism.

It should also be taken into consideration that inflammation is involved in the development of cardiovascular complications in SHR rats [44,45,46]. Plasma levels of proinflammatory IL-1β and IL-6 were found as significantly higher in SHR rats when compared with normotensive WKY rats [44]. The inflammation is accompanied by various biophysical changes in RBCs [47]. So, the different effects of USPION administration on RBCs of WKY rats and SHR rats could be at least partially ascribed to differences in the inflammatory status of the experimental animals.

## 5. Conclusions

Our study brought original results regarding the effect of single and repeated USPION administration on RBC properties under conditions of normal and high blood pressure in rats. Our findings showed elevated RBC deformability in USPION-treated WKY rats in both experimental designs without significant sights of either worsened RBC function or damage to the organs (investigated by determination of biochemical markers in plasma) in both WKY rats and SHR rats. We also found elevated NO production by RBCs after a single infusion of USPIONs. Finally, we observed that the accumulation of USPIONs in RBCs in hypertensive rats is much lower compared with the normotensive rats, which may be relevant for clinical practice in hypertensive patients undergoing clinical examination with the use of iron-oxide nanoparticles.

## Figures and Tables

**Table 1:** Biochemical parameters were determined in plasma after a single administration of USPIONs at the dose of 1 mg Fe/kg of body weight. Rats were killed approximately 100 min after the USPION administration. Abbreviations: USPIONs, ultra-small superparamagnetic iron-oxide nanoparticles; WKY, Wistar Kyoto rats; SHR, spontaneously hypertensive rats; AP, alkaline phosphatase; ALT, alanine aminotransferase; LDH, lactate dehydrogenase; AST, aspartate aminotransferase; P, phosphate. Results are presented as mean ± SD. * *p* < 0.0001 vs. WKY control; ^+^ *p* < 0.0001 vs. WKY USPIONs.

	Experiment 1						
Parameter	WKY		SHR		Two-Way ANOVA		
	Control	USPIONs	Control	USPIONs	Strain	Intervention	Interaction
Calcium (mmol/L)	2.5 ± 0.1	2.4 ± 0.1	2.3 ± 0.2	2.4 ± 0.1	ns	ns	ns
Potassium (mmol/L)	6.2 ± 1.6	5.8 ± 1.0	5.8 ± 0.4	5.9 ± 0.5	ns	ns	ns
Magnesium (mmol/L)	0.93 ± 0.08	0.96 ± 0.14	0.88 ± 0.04	0.87 ± 0.12	ns	ns	ns
Sodium (mmol/L)	143.3 ± 1.8	143.6 ± 2.5	140.8 ± 2.5	140.0 ± 2.3	F_(1,20)_ = 10.4 *p* < 0.005	ns	ns
AP (U/L)	106.0 ± 7.4	94.3 ± 12.5	98.2 ± 16.7	110.9 ± 10.8	ns	ns	F_(1,22)_ = 4.47 *p* < 0.05
ALT (U/L)	29.1 ± 7.6	29.3 ± 4.5	31.0 ± 4.7	29.1 ± 5.6	ns	ns	ns
LDH (U/L)	1092 ± 743	1124 ± 662	1338 ± 434	1124 ± 328	ns	ns	ns
AST (U/L)	90.2 ± 53.6	69.1 ± 19.6	116.7 ± 35.1	99.85 ± 21.6	ns	ns	ns
Cholesterol (mmol/L)	2.7 ± 0.4	2.8 ± 0.4	1.3 ± 0.1 *	1.5 ± 0.2 ^+^	F_(1,21)_ = 10.75 *p* < 0.0001	ns	ns
Triglycerides (mmol/L)	0.54 ± 0.15	0.91 ± 0.45	0.44 ± 0.11	0.67 ± 0.31	ns	F_(1.21)_ = 5.39 *p* < 0.05	ns
Uric acid (µmol/L)	37.8 ± 4.4	47.0 ± 20.1	61.5 ± 14.8	46.6 ± 14.5	ns	ns	ns
Creatinine (µmol/L)	31.5 ± 3.0	32.0 ± 5.1	29.3 ± 2.4	28.3 ± 0.5	ns	ns	ns
Inorg. P (mmol/L)	2.40 ± 0.14	2.36 ± 0.20	2.35 ± 0.33	2.14 ± 0.27	ns	ns	ns
Total proteins (g/L)	58.8 ± 5.6	57.7 ± 4.6	55.5 ± 5.8	55.7 ± 4.3	ns	ns	ns
Albumins (g/L)	39.3 ± 3.3	38.7 ± 3.2	37.4 ± 3.6	39.4 ± 3.5	ns	ns	ns
Urea (mmol/L)	6.2 ± 0.5	6.6 ± 1.2	6.1 ± 1.1	5.8 ± 0.5	ns	ns	ns

**Table 2:** Biochemical parameters determined in plasma after double administration of USPIONs at a dose of 2 mg of Fe/kg of body weight per day. Rats were killed approximately 24 h after the second USPION administration. Abbreviations: USPIONs, ultra-small superparamagnetic iron-oxide nanoparticles; WKY, Wistar Kyoto rats; SHR, spontaneously hypertensive rats; AP, alkaline phosphatase; ALT, alanine aminotransferase; LDH, lactate dehydrogenase; AST, aspartate aminotransferase; P, phosphate. Results are presented as mean ± SD. * *p* < 0.05, *** *p* < 0.0001 vs. WKY control; ^+^ *p* < 0.01, ^++^ *p* < 0.005; ^+++^ *p* < 0.001 vs. WKY USPIONs; ^#^ *p* < 0.01; ^###^ *p* < 0.0001 vs. SHR controls.

	Experiment 2						
Parameter	WKY		SHR		Two-Way ANOVA		
	Control	USPIONs	Control	USPIONs	Strain	Intervention	Interaction
Calcium (mmol/L)	2.6 ± 0.04	2.6 ± 0.1	2.6 ± 0.1	2.6 ± 0.1	ns	ns	ns
Potassium (mmol/L)	5.6 ± 0.4	5.2 ± 0.3	5.6 ± 0.4	5.8 ± 0.6	ns	ns	ns
Magnesium (mmol/L)	1.03 ± 0.13	0.94 ± 0.05	0.97 ± 0.04	0.95 ± 0.05	ns	ns	ns
Sodium (mmol/L)	141.3 ± 1.1	140.3 ± 1.1	140.4 ± 1.1	140.6 ± 1.3	ns	ns	ns
AP (U/L)	94.0 ± 14.0	93.3 ± 7.8	113.0 ± 4.2 *	113.9 ± 12.4 ^+^	F_(1,24)_ = 24.48 *p* < 0.0001	ns	ns
ALT (U/L)	34.76 ± 5.5	44.7 ± 23.0	35.9 ± 9.7	30.8 ± 7.9	ns	ns	ns
LDH (U/L)	926 ± 143	960 ± 133	970 ± 142	1036 ± 232	ns	ns	ns
AST (U/L)	93.3 ± 15.3	100.5 ± 36.2	89.7 ± 11.3	85.8 ± 16.6	ns	ns	ns
Cholesterol (mmol/L)	2.9 ± 0.5	2.8 ± 0.2	1.8 ± 0.1 ***	1.7 ± 0.2 ^+++^	F_(1,26)_ = 88.69 *p* < 0.0001	ns	ns
Triglycerides (mmol/l)	1.27 ± 0.16	1.42 ± 0.36	1.14 ± 0.13	1.11 ± 0.21	F_(1,26)_ = 7.025 *p* < 0.05	ns	ns
Uric acid (µmol/L)	38.9 ± 7.0	32.4 ± 6.2	87.5 ± 22 ***	47.6 ± 3.5 ^###^	F_(1,26)_ = 49.14 *p* < 0.0001	F_(1,26)_ = 25.85 *p* < 0.0001	F_(1,26)_ = 13.48 *p* < 0.0001
Creatinine (µmol/L)	31.4 ± 4.4	27 ± 1.3 *	31.6 ± 1.3	27.0 ± 1.6 ^#^	ns	F_(1,25)_ = 23.57 *p* < 0.0001	ns
Inorganic P (mmol/L)	2.37 ± 0.21	2.37 ± 0.05	2.16 ± 0.20	2.03 ± 0.15 ^++^	F_(1,25)_ = 19.27 *p* < 0.0005	ns	ns
Total proteins (g/L)	63.3 ± 3.8	59.6 ± 5.8	62.2 ± 1.9	62.8 ± 2.5	ns	ns	ns
Albumins (g/L)	40.7 ± 1.6	37.6 ± 3.7	41.6 ± 1.6	40.7 ± 1.7	F_(1,26)_ = 5.657 *p* < 0.05	F_(1,26)_ = 5.947 *p* < 0.05	ns
Urea (mmol/L)	7.0 ± 1.2	7.3 ± 1.6	8.4 ± 1.2	7.7 ± 0.7	ns	ns	ns

## Data Availability

The data presented in this study are available in this manuscript.
